# A Systematic Review of Cellulitis Guidelines: The Role of Non‐Pharmacological Management in Preventing Recurrence

**DOI:** 10.1111/ajd.14546

**Published:** 2025-06-18

**Authors:** Janette Jacka, Robyn Sierla, Sharon L. Kilbreath, Elizabeth S. Dylke

**Affiliations:** ^1^ Sydney School of Health Sciences, Faculty of Medicine and Health The University of Sydney Sydney Australia; ^2^ Department of Occupational Therapy Royal Prince Alfred Hospital Sydney Australia

## Abstract

Cellulitis is a common acute skin and soft tissue infection with high rates of recurrence. Historically, guidelines for care have focused on pharmacological management; however, emerging evidence supports the benefits of non‐pharmacological approaches for risk mitigation and recurrence prevention. This review examined best practice guidelines for the management of cellulitis—for the inclusion of recommendations beyond antibiotics to prevent recurrence. A systematic search of seven databases was conducted, including the Guidelines International Network (GIN), the Agency for Healthcare Research and Quality and the Australian National Health and Medical Research Council clinical practice guidelines. Guidelines were included if they addressed lower limb cellulitis, were developed after 2000, published in English, appeared in a peer‐reviewed journal and/or were endorsed by a health agency or government body, and contained recommendations for non‐pharmacological management. The Appraisal of Guidelines for Research and Evaluation II (AGREE II) tool was used to assess guideline quality. Ten guidelines met the inclusion criteria. Recommendations for non‐pharmacological management and recurrence prevention primarily addressed risk factors such as venous disease, oedema, tinea, skin barrier integrity and obesity. However, these recommendations were often brief and lacked actionable strategies for managing local risk factors effectively. Most guidelines scored poorly in the AGREE II assessment, particularly in rigour of development, despite performing well in scope, purpose and clarity. Furthermore, non‐pharmacological strategies and recurrence prevention remain underdeveloped. Strengthening the evidence base and incorporating targeted prevention strategies will be essential to improving future guidelines, ultimately enhancing patient outcomes and reducing the burden on healthcare systems.

**Trial Registration:** Registered with Open Science Framework OSF: osf‐registrations‐7wkvf‐v1

## 1 Introduction

Cellulitis is a commonly occurring acute skin infection that occurs when a pathogen gains entry into the dermis through a break in the skin [1]. It presents as redness, swelling and warmth over the affected area and is accompanied by pain, fever and fatigue. Severity ranges from localised erythema to rapidly spreading infection and sepsis. Rates of cellulitis are high; in 2017–2018, it was the fourth most common potentially preventable reason for admission to hospital in Australia, with rates of hospitalisation of 256 per 100,000 [2]. This creates a significant burden on the health care system [3, 4].

Each episode of cellulitis increases the likelihood of another [2, 5]. Globally, rates of recurrence for cellulitis range between 22% and 49%, with subsequent infections often more severe, resulting in longer hospital stays and increased pressure on the health care system [3, 5, 6]. Traditionally, guidelines and practice recommendations focus on the pharmacological management of the acute episode, with antibiotics and prophylactic antibiotics used to address both the acute episode and recurrence [7, 8, 9, 10, 11]. Prophylactic antibiotics reduce recurrence by 56%, but their effect ends when treatment stops [12]. Additionally, prophylactic antibiotic use is controversial due to antimicrobial stewardship, and their use should be considered along with the wider management of risk factors for recurrence [1, 13].

Common comorbidities and risk factors for cellulitis are well‐known, and growing evidence supports managing modifiable risk factors to reduce recurrence (secondary prevention) [3, 14, 15]. The main risk factors for cellulitis recurrence are chronic oedema and tinea pedis [1, 12]. Addressing these risk factors may provide an avenue for reducing recurrences of cellulitis. For example, recent studies investigating the impact of treatment of oedema on recurrent cellulitis provide compelling evidence that the management of this risk factor can have a major effect on reducing cellulitis incidence [4, 15]. Given this emerging evidence, it is timely to assess whether current cellulitis guidelines include recommendations for managing risk factors to prevent recurrence [4, 15, 16]. Due to the importance of guidelines in guiding clinical practice, if this emerging evidence is not included in guidelines, this may result in missed opportunities for recurrence prevention.

The aim of this review, therefore, is to describe current recommendations beyond the pharmacological management of cellulitis with the goal to reduce recurrence. In addition, the quality of the guidelines will be evaluated.

## 2 Materials and Methods

### 2.1 Search Strategy

**FIGURE 1:**
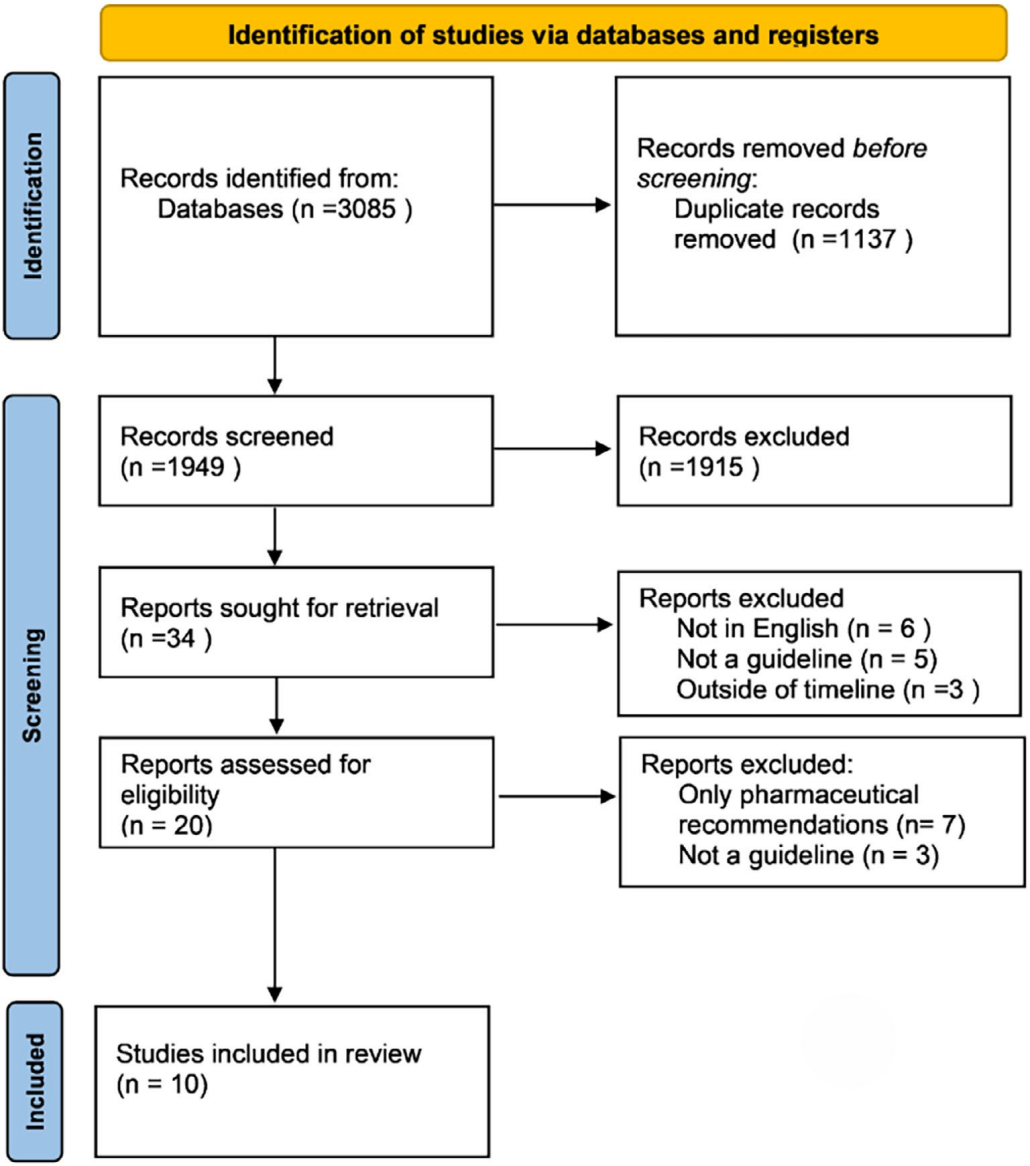
PRISMA 2020 flow diagram. APA, American Psychological Association; DLQI, Dermatology Life Quality Index; RCT, randomised controlled trial.

The literature was systematically reviewed to identify guidelines for the management of cellulitis (Figure 1: PRISMA flow diagram). Search terms for electronic databases used the concepts of cellulitis, clinical practice guidelines and non‐pharmacological management, for example rehabilitation, conservative, therapy. Five databases were searched: Medline, Embase, CINAHL, SCOPUS and BMJ. A further search of specific guideline databases, including Guidelines International Network (GIN), Agency for Healthcare Research and Quality and Australian National Health and Medical Research Council clinical practice guidelines, was conducted using the terms identified for cellulitis. These databases were chosen as they covered the spectrum of research areas that may be looking at cellulitis prevention. The search was undertaken in June 2021, with a further search undertaken in May 2023 and September 2024.

### 2.2 Article Selection

To be included in this review, guidelines had to be published in English in a peer‐reviewed journal and/or endorsed by a national/multinational government agency or health professional provider group strategies and be published between 2000 and 2024. In addition, the guidelines had to include recommendations for adults on lower limb cellulitis and include non‐pharmacological management. Guidelines for paediatric and head and neck cellulitis were excluded. For this review, non‐pharmacological management strategies were defined as those not requiring medication and could include management outside of the acute management phase or prophylactic antibiotic phase.

The search identified 3085 titles, of which 1137 were duplicates. Following screening of titles and abstracts screened independently by three members of the team, 20 full papers were reviewed by two members of the team. Ten papers were retained for inclusion in the review.

Any discrepancies in article inclusion were discussed by two members of the research team to reach consensus. Fewer than two discrepancies in article inclusions occurred. These included guidelines references that were then hand searched to identify any further guidelines for screening or inclusion; however, none were found.

Quality of the best practice guidelines was evaluated using the Appraisal of Guidelines for Research and Evaluation II (AGREE II). This tool assesses and quantifies guideline bias and provides insight on the rigour of the guideline development process [17], thereby providing an appraisal for the current guidelines' methodological development.

### 2.3 Data Extraction

Data were extracted independently by three members of the research team, with each guideline assessed by two team members independently. Data extracted included year, country, organisation, aim, endorsing body and types of recommendations. Only the recommendations for non‐pharmacological management and prevention of recurrence were summarised.

### 2.4 Critical Appraisal of Guidelines

The guidelines were assessed using the Appraisal of Guidelines for Research and Evaluation (AGREE II). The AGREE II consists of six domains, 23 items and an overall rating. Each item is scored from one to seven, with a score of one indicating that the item was poorly reported and seven indicating the item was well represented [17]. The domains include (1) scope and purpose, (2) stakeholder involvement, (3) rigour of development, (4) clarity of presentation, (5) applicability and (6) editorial independence. The tool also has two overall assessment items, which the appraiser makes an overall judgement about the guideline.

Each guideline was assessed using the AGREE II by two appraisers independently [17]. Each appraiser was provided with the AGREE II and user manual and familiarised themselves with the tool prior to analysing the guidelines. Further review of scores was conducted if there was any major discrepancy of more than four between each rating of an item. Any discrepancies were resolved with independent review of scores and discussion between the appraisers.

The scores were scaled for each domain as per the AGREE II to provide an average score domain. This involves calculating and summarising the scores for each domain into a standardised percentage of the maximum score that can be obtained for that domain [17]. As per the guidelines [17], discussion between appraisers was undertaken to obtain a consensus on the scaled score percentage to be used to differentiate between high‐quality and poor‐quality guidelines. A score of > 60% was used in several health‐based systematic reviews to indicate satisfactory quality [18, 19, 20] and so was chosen as the threshold for satisfactory quality for this review.

## 3 Results

Database searches returned 3085 results from which 1137 duplicates were removed. Following the review of the titles and abstracts, 20 full‐text publications were screened. Three publications were excluded as they were not in English, and seven were not included due to a lack of non‐pharmacological recommendations, resulting in 10 guidelines that met all inclusion criteria. Of the specific guideline database only, the International Guidelines Library (GIN) yielded one result, which had already been included. Hand searching of included guideline references elicited no additional results. The second search elicited one updated guideline; the third, no further guidelines. Guidelines were from a variety of countries through Europe [9, 10, 21, 22, 23], Asia [24] and America [25, 26]. Seven of the guidelines [9, 10, 11, 21, 23, 24, 25] were consensus documents and were developed by a large panel of experts in various fields including lymphoedema, infectious diseases, dermatology and surgery. Three of the guidelines were extracted from guideline repositories [9, 10, 11] and the remaining seven were published as journal articles.

Data extracted from the guidelines is summarised in Table 1. Recommendations of non‐pharmacological management strategies were limited in all guidelines. These recommendations are summarised in Table 2. The most common recommendations were compression for oedema [10, 25, 27], education around symptoms and prevention [9, 29, 30], elevation of the affected area including bed rest [9, 10, 11, 24, 28, 30] and practitioners' management of the underlying risk factors [9, 10, 11, 21, 22, 24, 25, 27, 28, 29, 30]. The recommendations varied in specificity.

**TABLE 1:** Summary of included guidelines.

Endorsing body	Title of guideline	Authors	Country of origin	Year of publication	Target audience
British Lymphology Society and the Lymphoedema Support Network' UK [10]	Consensus document on the management of cellulitis in lymphoedema	Consensus group: V. Keeley, PS Mortimer, A Hughers LSN Trustees, K Riches	United Kingdom	2022	Allied health
Wounds UK [8]	Management guidelines for lower limb cellulitis	Beldon P, Burton F	United Kingdom	2005	Nurse practitioners
Unknown [27]	Consensus conference on Erysipelas and necrotising fasciitis management. Short text	Anonymous	France	2000	Clinicians
Infectious Diseases Society of America [28]	Practice guidelines for the diagnosis and management of skin and soft tissue infections: 2014 update by the Infectious Diseases Society of America	Update of 2005 guidelines Dennis L. Stevens, Alan L. Bisno, Henry F. Chambers, E. Patchen Dellinger, Ellie J. C. Goldstein, Sherwood L. Gorbach, Jan V. Hirschmann, Sheldon L. Kaplan, Jose G. Montoya, and James C. Wade	USA	2014	Physicians
German Society of Dermatology [22]	Frequent bacterial skin and soft tissue infections: diagnostic signs and treatment	Sunderkotter, Cord; Becker, Karsten	Germany	2015	Health practitioners managing cellulitis
The Korean Society of Infectious Diseases [24]	Clinical guidelines for the antibiotic treatment for community‐acquired skin and soft tissue infection	Kwak, Yee Gyung; Choi, Seong‐Ho; Kim, Tark; Park, Seong Yeon; Seo, Soo‐Hong; Kim, Min Bom; Choi, Sang‐Ho	Korea	2017	General practitioners, residents and specialists responsible for inpatient, outpatient and emergency room care in medical institutions
External reviewers for ‘Geriatric Nursing’ [29]	Gerontologic nurse practitioner care guidelines: cellulitis in the elderly person	Wiliams J, Schmidt Luggen A	USA	2004	Gerontologic nurse practitioners
Central Medical Advisory Committee in 1988, Northern Ireland [9]	Guidelines on the management of cellulitis in adults (CREST, 2005)	Multidisciplinary sub‐group of health care professionals Chaired by Dr. Raymond Fulton	Northern Ireland	2005	Health services—primary care and secondary care
Italian Society of Infectious Tropic Disease and Int'l Society of Chemotherapy [21]	Diagnosis and management of skin and soft tissue infections (SSTI). A literature review and consensus statement: an update	Esposito et al. 2017	Italy	2017	Medical practitioners
Australasian Lymphology Association [11]	Management guidelines of cellulitis in lymphoedema	Members of the ALA council	Australia	2015	Those treating people with lymphoedema and cellulitis

**TABLE 2:** Summary of guidelines non‐pharmacological recommendations.

Types of non‐pharmacological recommendations	
Education	Patients be provided with education regarding recurrence, prevention and signs and symptoms [9, 29, 30] Patients be provided with information sheets [30]
Elevation/bed rest	Patients should be encouraged to elevate the affected area [9, 10, 11, 24, 25, 30]
Address risk factors	Prevention of recurrence involves management of risk factors [11, 24]. Risk factors which may require management: ‐ Tinea [10, 22, 25, 27, 29] ‐ Oedema [9, 11, 21, 25, 27, 29], managed with bedrest [9, 10, 30], decongestive lymphatic therapy [10], compression bandages and/or garments where tolerated [9, 10, 11, 24, 25, 27], sequential pneumatic compression pump [25] ‐ Venous disease [25, 27, 30] ‐ Obesity [10, 11, 24, 25] ‐ Wounds [9, 10] ‐ Surgery [10] ‐ Diabetes [11] ‐ Address skin damage [10, 11, 21, 22, 24, 25, 27]
Encourage mobility	Encourage mobility [10, 11, 30]

### 3.1 Methodological Quality of Guidelines

Ratings for the practice guidelines reviewed with the AGREE II and their scores across the domains are summarised in Table 3. There were discrepancies between appraisers of ≥ 4 for nine items. The appraisers reviewed their scores independently after recalibrating their shared understanding of the criteria and resubmitted their scores. Following this process, all scores were within four points.

**TABLE 3:** AGREE II guidelines for management of cellulitis: quality assessment.

Scores	AGREE II domain					
Guideline	Scope and purpose	Stakeholder involvement	Rigour of development	Clarity of presentation	Applicability	Editorial independence
Consensus document on the management of cellulitis in lymphoedema BLS 2022	92%	72%	81%	100%	71%	33%
Management guidelines for lower limb cellulitis Beldon 2005	95%	36%	14%	81%	63%	42%
Consensus conference on erysipelas and necrotising fasciitis management. Short text. Anon 2000	36%	0%	1%	31%	29%	0%
Practice guidelines for the diagnosis and management of skin and soft tissue infections: 2014 update by the Infectious Diseases Society of America	97%	58%	88%	89%	71%	96%
Frequent bacterial skin and soft tissue infections: diagnostic signs and treatment Sundkotter 2015	86%	36%	25%	92%	25%	75%
Clinical guidelines for the antibiotic treatment for community‐acquired skin and soft tissue infection Kwak 2017	92%	53%	73%	83%	35%	88%
Gerontologic nurse practitioner care guidelines: cellulitis in the elderly person Williams 2004	64%	33%	13%	72%	31%	21%
Guidelines on the management of cellulitis in adults (CREST, 2005)	61%	50%	22%	78%	63%	46%
Diagnosis and management of skin and soft tissue infections (SSTI). A literature review and consensus statement: an update Esposito 2017	69%	25%	0%	92%	25%	50%
Management of cellulitis in lymphoedema Australasian Lymphology Association 2015	94%	47%	63%	89%	48%	83%

Scheduled updates of guidelines are an important part of the AGREE II. It is generally recommended that health guidelines are updated every 3–5 years. However, the process for updating the guidelines to include current evidence was not well defined. Only five guidelines had been updated in the period 2000–2024 [10, 11, 21, 22, 25] with all updates undertaken in the last 7 years. In addition, only three guidelines stated a timeframe for updating or review [10, 24, 25]. The guideline most recently updated was by the British Lymphology Society, which was updated in 2022; the updated version scored significantly higher than the previous version in the domains of rigour of development and editorial independence.

Overall, the guidelines performed poorly on many of the AGREE II domains (Table 3). Only four guidelines scored above 60% on the rigour of development domain [10, 11, 24, 25]. Four guidelines received scores of over 60% in four domains [10, 11, 24, 25] with only three [10, 11, 24] scoring > 80% in three domains. The area in which most guidelines scored lowest was in rigour of development, in which appraisers' scores reflected unsatisfactory inclusions of search methods, evidence selection criteria, strengths and limitations of evidence, formulations of recommendations, considerations of benefits and harm, link between recommendations and evidence, external review and updating procedure [21, 22, 27, 29, 30]. Overall, the included guidelines performed best in Domain 1: scope and purpose and Domain 4: clarity of presentation, in which the majority scored above 80%.

## 4 Discussion

This systematic review investigated guidelines for the management and prevention of recurrence of cellulitis, beyond antibiotics. While a pharmacological approach to cellulitis treatment is necessary, secondary prevention by managing modifiable risk factors [4, 15] significantly reduces recurrence rates. The most commonly cited additional strategies for the management of cellulitis were recommendations around the use of compression [9, 10, 24, 25, 30] and elevation of the affected area [9, 10, 11, 24, 25, 27], both to address the swelling that frequently occurs in people who have contracted cellulitis. In addition, education around symptoms and prevention [9, 29, 30] and decongestive therapy (which aims to reduce oedema) [10, 24, 27] were suggested. Other recommendations highlighted the need for management of modifiable risk factors which likely contribute to recurrent cellulitis [11, 24]. These include tinea [10, 22, 25, 27, 29], oedema [9, 10, 11, 21, 25, 27, 29], venous disease [25, 27, 30], skin barrier breakdown [10, 11, 21, 24, 27], obesity [10, 11, 24, 25], wounds [9, 10], decreased mobility [11, 30] and diabetes [31].

Use of non‐pharmacological approaches for management of underlying risk factors associated with recurrent cellulitis can be highly effective. As such, highlighting alternative management approaches and including risk factor management in best practice guidelines has growing importance. For example, in a randomised controlled trial in which oedema was managed through the use of compression, recurrent cellulitis was significantly reduced to 15% risk compared to 40% with education alone [4]. In support, another study demonstrated the control of swelling was strongly associated with a significantly low risk of cellulitis [15]. Of the guidelines reviewed, only two [10, 11] provided sufficiently detailed strategies to address comorbidities such as oedema. In these two guidelines, evidence supporting non‐pharmacological management of predisposing conditions such as oedema, obesity, eczema, venous insufficiency and toe web abnormalities were reported as weak [24, 25]. The lack of robust evidence on the effects of managing risk factors is likely a contributing factor to the reduced focus placed on non‐pharmacological management of cellulitis. Further studies are required to explore the impact of managing strong risk factors such as tinea or other fungal infections on the occurrence and recurrence of cellulitis [12].

Broadly, the guidelines in this review were developed by medical specialists. The panels of experts consisted of infectious disease specialists, dermatologists, surgeons and specialist nurse practitioners [10, 21, 22, 24, 25, 26, 29, 30]. As the guidelines were developed with a predominantly medical focus in which the emphasis was on the pharmacological management, the lack of attention to issues that are not managed pharmacologically is not surprising. Guidelines in which the panel members were drawn from the broadest multidisciplinary backgrounds provided the most specific and detailed non‐pharmacological recommendations with greater focus on prevention of recurrent cellulitis [9, 10, 11]. For example, the British Lymphology Society guidelines [10] provided the most specific non‐pharmacological recommendations. This guideline was developed with consensus from a multidisciplinary panel that included experts from nursing, medical (including lymphoedema and dermatology) and members of a lymphoedema support network. It is only by this multi‐disciplinary approach that addresses the acute infection as well as known risk factors that the rates of recurrence are likely to decrease.

The review also aimed to examine the quality of development underpinning the guidelines. Most guidelines did not adhere to the processes recommended for the development of guidelines, such as those assessed by the AGREE II tool, to ensure high quality, nonbiased guidelines that are developed with appropriate consumer involvement. While some followed structures like GRADE [24, 25] which provides a framework to enable the development of high quality nonbiased guidelines [31], others were written as expert opinions [9, 30]. The literature selection in many of the included guidelines did not appear to be based on systematic review [9, 10, 11, 27, 29, 30] and so it is unclear how they determined which literature was included. A systematic approach to reviewing the literature informing the guidelines is needed. Greater rigour is necessary in the creation of these practice guidelines, including measurable review periods ensuring that new evidence can be incorporated. Future guidelines should provide more detailed information regarding their development process, according to AGREE II guidelines, to ensure quality guidelines are formed.

In addition to the vigorous development process for guidelines, a regular process for review is required. Only five of the ten included guidelines were updated within the last 5–10 years [10, 11, 21, 22, 25]. Of interest, the guideline which was most recently updated, in 2022 [10], also provided the most comprehensive recommendations around management of modifiable risk factors. Without review, guidelines can not encompass emerging evidence, thus limiting their ability to broaden the scope of evidence‐based recommendations and management strategies, which is what best practice guidelines should represent. Overall, guidelines need better processes to develop them rigorously and more research is needed to investigate how best to prevent recurrent cellulitis which can feed into future guidelines.

## 5 Conclusion

This review highlights the limited number of well‐developed guidelines with non‐pharmacological recommendations for cellulitis management. The results indicated that while most guidelines mentioned the management of risk factors such as oedema, skin infections and obesity, they lacked guidance on the specific interventions required to effectively mitigate these risk factors. As cellulitis is a leading cause of preventable hospital admissions, expanding guidelines to reflect the complex needs of at‐risk populations, alongside clear recommendations for recurrence prevention, could enhance patient outcomes and reduce healthcare system burden. While guidelines are now starting to include non‐pharmacological management strategies, more research is required in this area to support specific recommendations.

## Conflicts of Interest

The authors declare no conflicts of interest.

## Data Availability

The data that support the findings of this study are available from the corresponding author upon reasonable request.

## Appendix A Search Terms

Database: Ovid MEDLINE(R) ALL <1946 September 2024>

1. Cellulitis/or cellulitis.mp. (13564)
2. erysipelas.mp. or Erysipelas/ (2700)
3. 1 or 2 (15903)
4. Practice Guidelines as Topic/or Practice guideline*.mp. (166213)
5. protocol*.mp. (629683)
6. clinical pathway*.mp. (3892)
7. consensus.mp. or Consensus/ (191032)
8. expert opinion*.mp. (25540)
9. 4 or 5 or 6 or 7 or 8 (974361)
10. self management.mp. or Self‐Management/ (21889)
11. risk management.mp. or Risk Management/ (27613)
12. physiotherapy.mp. (21255)
13. therap*.mp. (6392504)
14. follow up.mp. (1399621)
15. prevent*.mp. (2491693)
16. Conservative Treatment/or conservative treatment*.mp. (35699)
17. Recurrence/or recurren*.mp. (719497)
18. 10 or 11 or 12 or 13 or 14 or 15 or 16 or 17 (9023019)
19. 3 and 9 and 18 (3085)
